# A Retrospective Analysis of the De Ritis Ratio in Muscle Invasive Bladder Cancer, with Focus on Tumor Response and Long-Term Survival in Patients Receiving Neoadjuvant Chemotherapy and in Chemo Naïve Cystectomy Patients—A Study of a Clinical Multicentre Database

**DOI:** 10.3390/jpm12111769

**Published:** 2022-10-27

**Authors:** Victoria Eriksson, Oscar Holmkvist, Ylva Huge, Markus Johansson, Farhood Alamdari, Johan Svensson, Firas Aljabery, Amir Sherif

**Affiliations:** 1 Department of Surgical and Perioperative Sciences, Urology and Andrology, Umeå University, 901 87 Umeå, Sweden; 2 Department of Clinical and Experimental Medicine, Division of Urology, Linköping University, 581 83 Linköping, Sweden; 3 Department of Surgery, Division of Urology, Sundsvall-Härnösand County Hospital, 856 43 Sundsvall, Sweden; 4 Department of Urology, Västmanland Hospital, 721 89 Västerås, Sweden; 5 Department of Statistics, Umeå School of Business, Economics and Statistics (USBE), Umeå University, 901 87 Umeå, Sweden

**Keywords:** urinary bladder neoplasms, neoadjuvant therapy, cystectomy, clinical decision rules, prognosis

## Abstract

Background: A high pre-treatment De Ritis ratio, the aspartate transaminase/alanine aminotransferase ratio, has been suggested to be of prognostic value for mortality in muscle-invasive bladder cancer (MIBC). Our purpose was to evaluate if a high ratio was associated with mortality and downstaging. Methods: A total of 347 Swedish patients with clinically staged T2-T4aN0M0, with administered neoadjuvant chemotherapy (NAC) or eligible for NAC and undergoing radical cystectomy (RC) 2009–2021, were retrospectively evaluated with a low ratio < 1.3 vs. high ratio > 1.3, by Log Rank test, Cox regression and Mann–Whitney U-test (MWU), SPSS 27. Results: Patients with a high ratio had a decrease of up to 3 years in disease-free survival (DFS), cancer-specific survival (CSS) and overall survival (OS) (*p* = 0.009, *p* = 0.004 and *p* = 0.009) and 5 years in CSS and OS (*p* = 0.019 and *p* = 0.046). A high ratio was associated with increased risk of mortality, highest in DFS (HR, 1.909; 95% CI, 1.265–2.880; *p* = 0.002). No significant relationship between downstaging and a high ratio existed (*p* = 0.564 MWU). Conclusion: A high pre-treatment De Ritis ratio is on a population level, associated with increased mortality post-RC in endpoints DFS, CSS and OS. Associations decrease over time and require further investigations to determine how strong the associations are as meaningful prognostic markers for long-term mortality in MIBC. The ratio is not suitable for downstaging-prediction.

## 1. Introduction

In 2020, 573,000 people were diagnosed with urinary bladder cancer (UBC), making it the 10th most common cancer worldwide and 6th most common in men [1]. The incidence varies between countries. Sweden has approximately 3200 new cases every year, with 700 deaths yearly. Men are three times more likely to be affected than women. At diagnosis, 70–75% are non-muscle-invasive bladder cancer (NMIBC; clinical stages Ta, T1 and Tis) and 20–25% are muscle-invasive bladder cancer (MIBC; clinical stages T2–T4) [2]. MIBC-patients have had a worse prognosis, with a five-year survival of less than 50% [3]. The addition of Cisplatinum-based neoadjuvant chemotherapy (NAC) prior to radical cystectomy (RC) has improved survival. However, if patients are non-responders to NAC, this may result in delayed RC and adverse events with decreased downstaging. Downstaging is considered a surrogate marker for overall survival [4,5].

The classical staging systems of clinical tumor-node-metastasis (cTNM) from the American Joint Committee on Cancer (AJCC) and the World Health Organization (WHO) classifications of high and low tumor grade, is routine in classifying and evaluating prognosis in MIBC-patients. Still, the classifications have less significance in predicting 5-year progression-free survival (PFS) [6]. Finding pre-treatment factors with the potential to predict aggressive disease and survival post-RC, would be useful [7]. A more precise evaluation of prognosis could also affect treatment selection prior to RC [8]. No clinical markers concerning prognosis in MIBC are available from a simple blood test at present [9].

However, the De Ritis ratio has recently been described as having prognostic value in multiple individual cancer forms, such as renal cell carcinoma (RCC), myeloma, colonic, pancreatic and urothelial cancer [8,10,11,12,13,14,15]. The ratio consists of the well-known circulating enzymes aspartate aminotransferase (AST) and alanine aminotransferase (ALT) [3,10], which are retrievable by blood test. The AST/ALT ratio was initially presented by De Ritis in 1957 as a tool to asses liver disease [16]. The underlying mechanism between the AST/ALT enzymes and cancer is not fully clear, but aminotransaminases are expressed by both cancerous and noncancerous cells and found in the liver, heart, skeletal muscle, and kidney [13]. UBC is considered a glucose-dependent malignancy, and it is known that AST plays an essential role in glycolysis, which leads to a potential condition in which increased glucose metabolism induced by cancer, known as the Warburg effect, exists with an increase in AST/ALT ratio. Thus, a potential correlation between a high ratio and cancer disease has been hypothesized [7,8,13,17,18].

Retrospective studies on MIBC have reported findings in which a high ratio was prognostic for inferior PFS, lesser cancer-specific survival (CSS) and lower overall survival (OS). Similar methods to establish a cut-off value were used, including ROC curve with a high De Ritis ratio if >1.1–1.5. The studies may have made deeper reflections regarding potential weaknesses in the statistical methods that established the cut-off level [3,7,8,10,12,19]. One study was based on a population where 30% had disseminated disease and there was no mention of NAC [19]. Another study had a mixed population of MIBC and NMIBC (non-MIBC) and only 18% of the patients received NAC. One of the most important factors for assessing long-term survival, lymph node status pre-treatment, was reported in a vague way, where it is not possible to determine whether it was overt cN+ or whether postoperative pN+ was intended [3]. One study presented survival predictions solely in OS [7], which may include, but is not limited to, death by other types of cancers than MIBC, noncancer-related death and death from treatment. Thus, many events in patients that were specifically unrelated to MIBC were included [20]. Two meta-analyses from 2020 concluded that further validation of the De Ritis is required and that previous retrospective studies from up to 2020, including UBC, were often single-centre and lacking a diverse study population such as patients of African or American origin [11].

We intended to evaluate a larger cohort solely on NAC-treated and NAC-eligible patients after the exclusion of pre-treatment disseminated disease, bacillus Calmette-Guérin (BCG)-resistant UBC and liver disease, as opposed to some previous studies that included patients with known lymph-node involvement. Thus, our aspiration was a cohort with potentially less bias due to high comorbidity and disseminated disease. The aim was to investigate if an association between a high De Ritis ratio > 1.3 and increased mortality existed, by evaluating endpoints in different time periods regarding survival outcomes post-RC and evaluating the proxy marker downstaging of primary tumor. To the best of our knowledge, downstaging has not yet been evaluated in this research area.

## 2. Materials and Methods

**Figure 1:**
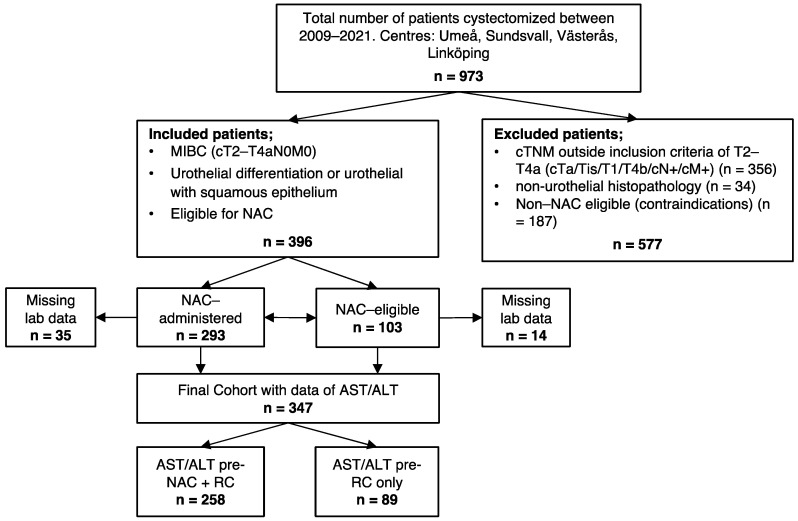
Flowchart displaying selection process of patient cohort, including inclusion and exclusion criteria. Muscle invasive bladder cancer (MIBC); clinical tumor node metastasis (cTNM); neoadjuvant chemotherapy (NAC); aspartate aminotransferase/alanine aminotransferase (AST/ALT); radical cystectomy (RC).

Study design and patient selection; Cystectomized patients were derived from an existing clinical, multi-centre database with all cystectomized patients between the years 2009 and 2021 (n = 973). The patients were treated at either four Swedish cystectomy centres; Norrlands Universitetssjukhus, Umeå; Länssjukhuset Sundsvall, Sundsvall; Västmanlands sjukhus, Västerås and Universitetssjukhuset Linköping, Linköping. Inclusion criteria were MIBC; cT2-T4aN0M0, urothelial histopathology and NAC-administered or NAC-eligible NAC-naïve patients. All patients were discussed after diagnosis at multidisciplinary conference and assigned NAC pre-RC if clinically acceptable. Exclusion criteria were liver disease, disseminated cancer, non-urothelial histopathology, and contraindications for NAC; i.e., age > 75, reduced kidney function, Charlson Age Comorbidity Index (CACI) > 6 and hearing impairment. Patients with missing data regarding AST/ALT, due to there being no available laboratory results, were excluded. Final analysis included 347 patients, all who had undergone RC (Figure 1).

Study procedure: clinicopathological variables from patient medical records were individually collected and compiled into an existing clinical database. Laboratory data were updated from 1 January 2009 to 31 January 2022. Variables included age, Body Mass Index (BMI), American Society of Anaesthesiologists (ASA) score, CACI, cTNM, pTNM, NAC or NAC-legibility, active smoker yes/no, outcome data on cancer recurrence and disease-free survival (DFS), CSS and OS. DFS was chosen instead of PFS as the presented endpoint in this study, with the included events initially described by Punt et al. [21] and later by Birgisson et al. [20]. AST/ALT was documented within 30 days pre-NAC or pre-RC, depending on the treatment the patients were assigned. AST and ALT were routinely tested in lithium heparin plasma with upper reference level 0.75 and 1.1 µ/L, respectively. De Ritis cut-off value in analysis was set to 1.3 based on a frequent used cut-off in previous studies [3,7,19]. Patients were categorized into two groups based on high ratio ≥ 1.3 or low ratio < 1.3. Downstaging of the primary tumor was divided into four different outcomes and ranked with values 1–4 on an ordinal scale; progressive disease (PD; pN+, pT4b), stable disease (SD; pT2–4aN0M0), partial response (PR; pTa, pTis, pT1N0M0) and complete response (CR; pT0N0M0). In analysis, variables on two levels were treated as nominal, such as age > 70 or age < 70, active smoker; yes or no, NAC; yes or no, ASA and pT-stage > 0; pT0 or pTis-T4b, considering the importance of pT0 as a survival benefit, according to Rosenblatt et al. [4]. The variables BMI and CACI were treated as interval variables.

Statistical analysis: Analysis on downstaging was performed separately on NAC-receivers, and the entire cohort of NAC and NAC-eligible NAC-naïve patients, to compare if the association with downstaging and a high ratio differed depending on the different NAC levels. The association between the pre-treatment De Ritis ratio and downstaging outcomes was evaluated by the Mann–Whitney U tests. Survival was predicted up to 3, 5 and 13 years with the study endpoints DFS, CSS and OS. The influence of the pre-treatment De Ritis ratio on the study endpoints was visualized and compared with the Kaplan–Meier estimator and the Log Rank test. Crude and adjusted Cox proportional regression analyses were carried out to determine the influence of patient gender, age, histological TN, pathological T stage, tumour grade and pre-treatment De Ritis ratios on DFS, CSS and OS. HRs estimated from Cox models are shown as the HR with the corresponding 95% CI. The Cox proportional hazard assumption were visually evaluated in STATA. All the used tests are two-sided with a significance level of 5%. The data were statistically evaluated in SPSS Statistics 27.0 for Mac (IBM Corporation, Armonk, NY, USA), and STATA version 15 (Stata Corp, Houston, TX, USA).

## 3. Results

The final cohort consisted of 347 patients (Figure 1). A total of 258 patients with NAC had pre-treatment data on AST/ALT and 89 NAC-eligible NAC-naïve patients had pre-treatment data on AST/ALT. A total of 76% of the total cohort were men, but a larger proportion of the women in the cohort existed within the high ratio group, compared to the low ratio group (Table 1, *p* = 0.044). A total of 35% of the patients in the cohort were aged over 70. Regarding comorbidity, median level of CACI was 5 and approximately 23% of 347 patients had ASA grade III. Additionally, 75% received some sort of NAC, of which 78% within the group of high ratios were NAC-receivers, compared to 73% NAC-receivers within the low-ratio group. The majority of the cohort-patients had initial stage cT2 (61%) (Table 1).

**Figure 2:**
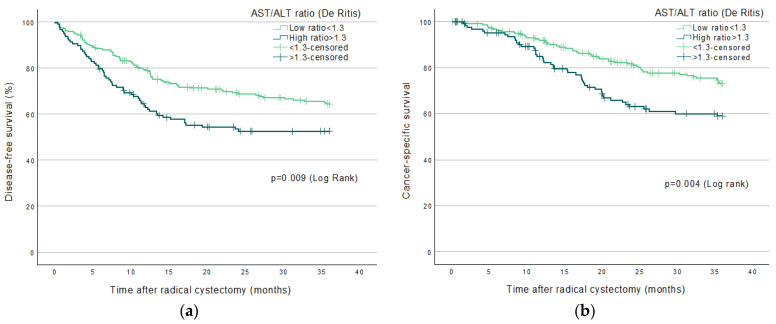

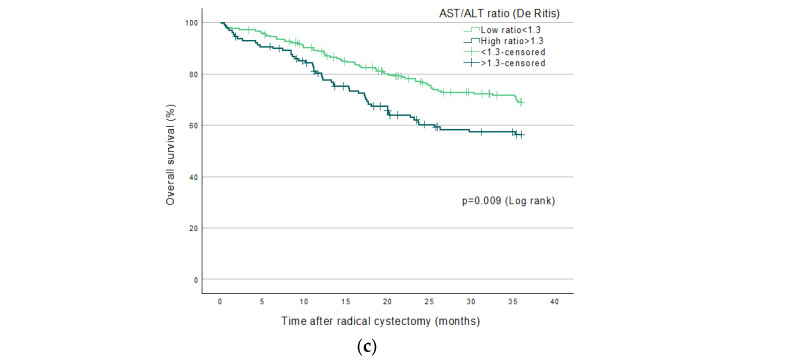
Kaplan–Meier curves predicting 36 (3 years) and 60 months (5 years) survival endpoints (**a**) Disease-free survival; (**b**) Cancer-specific survival; (**c**) Overall survival.

**Figure 3:**
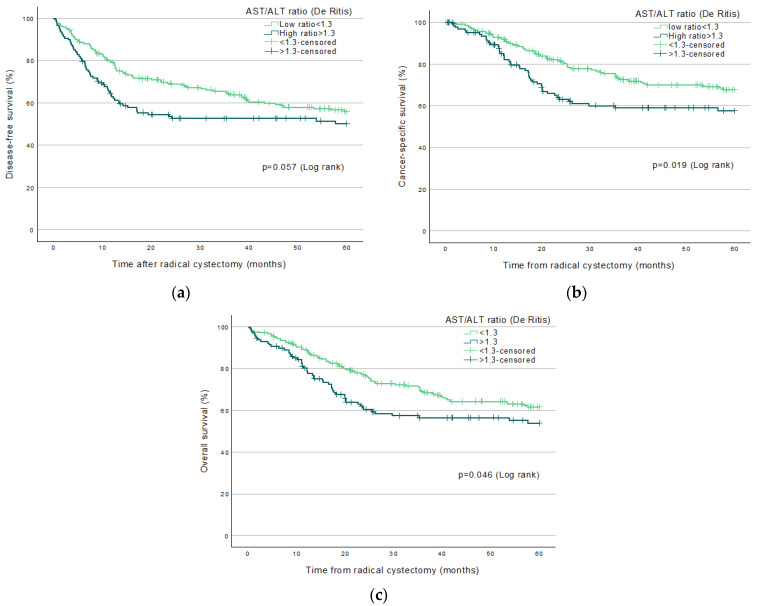
Kaplan–Meier curves predicting 60 months (5 years) survival endpoints (**a**) Disease-free survival; (**b**) Cancer-specific survival; (**c**) Overall survival.

**Figure 4:**
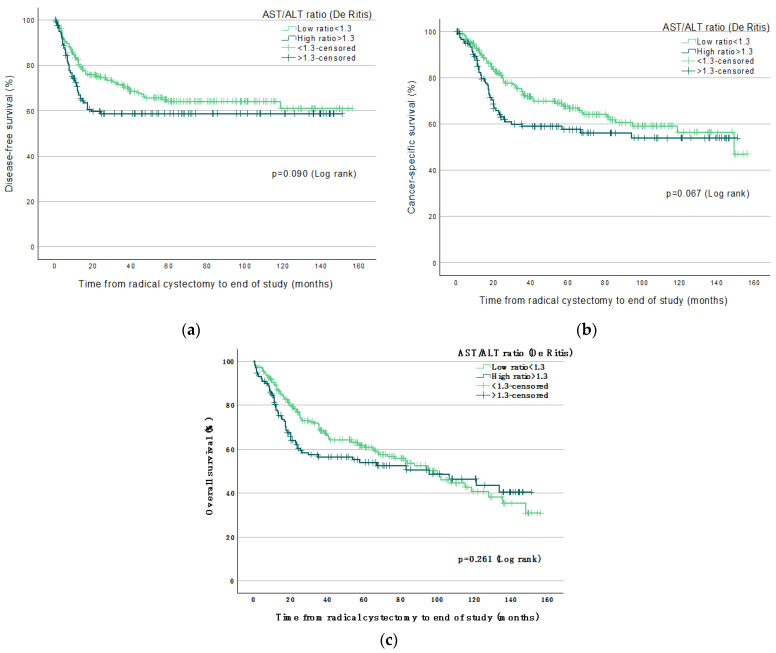
Kaplan–Meier curves predicting 5-13 years (160 months) post-RC survival endpoints (**a**) Disease-free survival; (**b**) Cancer-specific survival; (**c**) Overall survival.

Significantly increased mortality was found in patients with high De Ritis ratio; Log Rank-test, in endpoints DFS (*p* = 0.009), CSS (*p* = 0.004), and OS (*p* = 0.009) up to three years (36 months) post-RC (Figure 2a–c). Significantly increased mortality existed in patients with high De Ritis ratio in endpoints CSS (*p* = 0.019) and OS (*p* = 0.046) but not in DFS (*p* = 0.057) up to five years (60 months) post-RC (Figure 3a–c). No significant increased mortality existed in patients with high ratio, up to 13 years (160 months) post-RC in any endpoint; DFS (*p* = 0.090), CSS (*p* = 0.067), or OS (*p* = 0.261, Figure 4a–c).

Kaplan–Meier curves predicting survival endpoints up to 36 months.

Kaplan–Meier curves predicting survival endpoints up to 60 months.

Kaplan–Meier curves predicting survival endpoints up to 160 months.

The Cox proportional hazard assumption was visually evaluated and the hazard ratio (HR) between low and high De Ritis ratio seems constant up to three years, and then decreases somewhat. After five years, the proportional hazard assumption can no longer be considered valid. Hence, HR is only evaluated up to three and five years (Table 2). Regarding NAC, in neither crude nor adjusted Cox analysis can a significant interaction effect be visualized between a high De Ritis ratio and NAC, indicating that a high De Ritis ratio equally affects mortality in NAC-receivers and those who are NAC-eligible in our material. We used this as an argument to evaluate the effect of the De ritis ratio in the entire cohort.

A high De Ritis ratio > 1.3 was associated with increased risk of mortality in endpoints DFS, CSS and OS up to three years post-RC, up to five years in CSS and OS, both crude and adjusted for pT-stage > 0, age > 70, active smoking, ASA, CACI, BMI, and NAC. In addition, pT-stage > 0 was associated with increased risk of mortality in all endpoints up to three- and five-years post-RC when adjusted for ratio > 1.3, age > 70, active smoking, ASA, CACI, BMI, and NAC. No individual association was seen between mortality and the other variables age > 70, active smoking, ASA, BMI, and NAC, in any timeframe or endpoint (Table 2).

**Figure 5:**
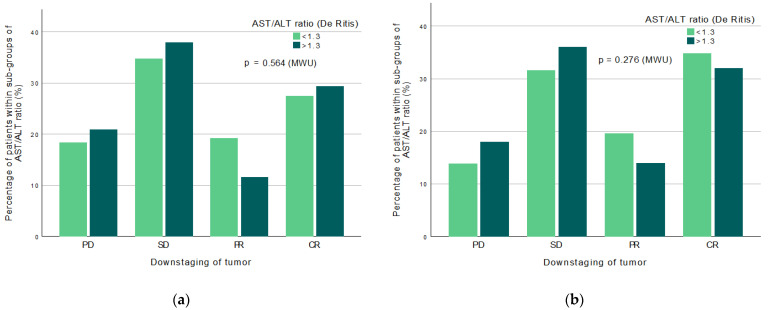
(**a**) Downstaging of primary tumor in patient cohort NAC and NAC-naïve n = 347, by the four downstaging categories with patients (%) within sub-groups of high or low De Ritis ratio. There is no significant relationship between downstaging and a high De Ritis ratio > 1.3 (*p* = 0.564 MWU); (**b**) Downstaging of primary tumor in NAC patients only n = 258, by the four downstaging categories with patients (%) within sub-groups of high or low De Ritis ratio. There is no significant relationship between downstaging and a high ratio > 1.3 (*p* = 0.276 MWU).

There is no significant relationship between the downstaging of primary tumor and a high De Ritis ratio > 1.3 (*p* = 0.564 MWU) either in the entire cohort (Figure 5a) or in NAC-receivers only (Figure 5b) (*p* = 0.276 MWU). A visible difference can be seen in distribution between the downstaging grades, where NAC-receivers have a higher percentage of increased downstaging within the survival markers of CR and PR (Figure 5b), compared to the entire cohort (Figure 5a).

Downstaging of tumor vs high AST/ALT ratio within all patients (left) and only NAC patients (right).

## 4. Discussion

A high pre-treatment De Ritis ratio was associated with higher mortality in both survival analysis (Figure 2a–c, Figure 3a–c and Figure 4a–c) and in crude and adjusted risk-analysis with increased HR if the ratio was >1.3 in patients, compared to low ratio <1.3. pT-stage >0 was also frequently associated with higher mortality in more endpoints than the De Ritis ratio (Table 2). Thus, a higher tumor stage with more severe cancer post-RC is most statistically associated with mortality than all of our other included variables, as was to be expected. We suggest that pT-stage post-RC that is of the current standard is still of higher prognostic value in predicting survival compared to a high De Ritis ratio pre-treatment. However, UBC is considered a glucose-dependent malignancy, and it is known that AST plays an essential role in glycolysis, which leads to a potential condition in which the increased glucose metabolism by cancer, known as the Warburg effect, exists with an increase in AST/ALT ratio and, therefore, possibly has a high ratio with cancer disease. However, the exact mechanism of the suggested condition has not been shown [8,13] and can only be hypothesized, which must be considered a weakness.

No significant relationship existed between downstaging of the primary tumor and a high De Ritis ratio in either the mixed cohort (*p* = 0.564 MWU) or in NAC-receivers only (*p* = 0.276 MWU) (Figure 5a,b). We, therefore, propose that the different NAC-levels did not affect the potential association between a high De Ritis ratio and downstaging, and that a high pre-treatment ratio may not be of prognostic value regarding downstaging response in either NAC- or NAC-eligible patients. To the best of our knowledge, these findings have not previously been described. The strength in this study is in the evaluation of a relatively large cohort (n = 347), considering extensive inclusion and exclusion criteria compared to other similar studies with higher heterogeneity in the study population. A selection bias could be argued regarding the selection process of patients described in the Methods, where patients with extra high comorbidity may have been excluded due to the inclusion criteria. However, our result may possibly reflect a healthier population than other, similar studies that made no such distinction between levels of disease pre-analysis. Consequently, we propose that our significant results are not primarily due to the high comorbidity in the study population, which may be considered a strength.

A potential weakness may be the HRs in Table 2. The Cox proportional hazard assumption was visually evaluated and the hazard ratio between low and high De Ritis ratio seems constant up to three years, and then decreases somewhat. After five years, the proportional hazard assumption can no longer be considered valid. Other unknown factors may influence the hazard around and after 3–5 years, which may be considered a weakness in the results displayed in Table 2. However, many recurrences were detected before three years post-RC, hence suggesting that the risk analysis utilizing De Ritis ratio has a clear interest for evaluation. Another weakness may lie in our cut-off level of a high De Ritis ratio > 1.3. This was selected from previous studies [3,7]. This may have influenced our results. However, we still had unique individual AST/ALT levels on our population, and can thus compare our results with studies that had the same cut-off level of 1.3, but different AST/ALT levels due to the different study population. We had similar results to several other studies regarding the association between higher mortality and a high ratio [7,10,19,22]. Finally, our results regarding an association between increased mortality and a high ratio lack statistical calculations regarding how strong that association may be. Therefore, we suggest that the strength in the association should be further investigated. This may provide more insight into whether said association between a high De Ritis ratio and mortality is so strong that the ratio can be considered a future prognostic factor for survival in MIBC.

## 5. Conclusions

A high pre-treatment De Ritis ratio is associated with an increased risk of mortality post-RC in endpoints DFS, CSS and OS. The association decreases over time and requires further investigation to determine how strong the association is as a long-term potential prognostic factor for mortality in MIBC. The De Ritis ratio is not suitable for predicting downstaging.

## Figures and Tables

**Table 1:** Patient cohort characteristics within groups of low and high De Ritis ratio and total cohort.

Variables	Low De Ritis Ratio < 1.3	High De Ritis Ratio ≥ 1.3	Total Cohort	*p*-Value
	n = 218	n = 129	n = 347	
Preoperative				
Sex, n (%)				
Male	173 (79.4)	90 (69.8)	263 (75.8)	0.044 *
Female	45 (20.6)	39 (30.2)	84 (24.2)	
Age, n (%)				0.055 *
<70 years	149 (68.3)	75 (58.1)	224 (64.6)	
>70 years	69 (31.7)	54 (41.9)	123 (35.4)	
BMI (mean, SD)	26.46 (3.9)	25.3 (3.9)	26.03 (3.9)	0.008 ^†^
ASA, n (%)				0.592 *
I	37 (17)	21 (16.3)	58 (16.7)	
II	134 (61.5)	74 (57.4)	208 (59.9)	
III	47 (21.6)	34 (26.4)	81 (23.3)	
CACI (median, IQR)	5 (4, 5)	5 (4, 5)	5 (4, 5)	0.275 ^ƒ^
Smoker, n (%)				0.966 *
Yes	57 (26.1)	34 (26.4)	91 (26.2)	
No	161 (73.9)	95 (73.6)	256 (73.8)	
cT-stage, n (%)				0.317 *
cT2	136 (62.4)	77 (59.7)	213 (61.4)	
cT3	73 (33.5)	41 (31.8)	114 (32.8)	
cT4a	9 (4.1)	11 (8.5)	20 (5.8)	
NAC, n (%)				0.299 *
Yes	158 (72.5)	100 (77.5)	258 (74.4)	
No	60 (27.5)	29 (22.5)	89 (25.6)	
NAC; MVAC, n (%)				0.521 *
Yes	131 (60.1)	82 (63.6)	213 (61.4)	
No	87 (39.9)	47 (36.4)	134 (38.6)	
Postoperative				
pT-stage n (%)				0.612 *
pT0	62 (28.4)	40 (31.0)	102 (29.4)	
pTCis-T4b	156 (71.6)	89 (69.0)	245 (70.6)	

* Chi-square test; ^†^ *t*-test; ^ƒ^ Mann–Whitney U-test; BMI; Body Mass Index, ASA; American Society of Anaesthesiologists, CACI; Charlson Age Comorbidity Index, cT2-4a-clinical-tumor-stage; NAC; neoadjuvant chemotherapy, pT0-T4b; pathological-tumor-stage.

**Table 2:** Cox analysis crude and adjusted on entire cohort; NAC and NAC-eligible NAC-naïve, n = 347, up to 3- and 5-years post-RC.

		Crude Estimates ^1^			Adjusted Estimates ^2^		
Type of Survival	Variables	HR	CI	*p*	HR	CI	*p*
Survival up to 3 years after RC							
DFS	AST/ALT > 1.3	1.573	1.117–2.215	0.010 *	1.673	1.175–2.380	0.004 **
	pT-stage > 0				2.654	1.628–4.325	0.000 **
CSS	AST/ALT > 1.3	1.772	1.191–2.636	0.005 *	1.909	1.265–2.880	0.002 **
	pT-stage > 0				3.913	2.059–7.435	0.000 **
OS	AST/ALT > 1.3	1.625	1.125–2.348	0.010 *	1.724	1.183–2.513	0.006 **
	pT-stage > 0				2.493	1.485–4.186	0.001 **
Survival up to 5 years after RC							
DFS	AST/ALT > 1.3	1.372	0.989–1.902	0.058	1.485	1.060–2.080	0.021 **
	pT-stage > 0				2.783	1.747–4.434	0.000 **
CSS	AST/ALT > 1.3	1.575	1.075–2.308	0.020 *	1.759	1.185–2.612	0.005 **
	pT-stage > 0				4.334	2.292–8.193	0.000 **
OS	AST/ALT > 1.3	1.427	1.005–2.025	0.047 *	1.556	1.086–2.228	0.016 **
	pT-stage > 0				2.564	1.567–4.194	0.000 **

^1^ Crude estimates of the De Ritis ratio < 1.3 or ratio > 1.3; ^2^ Adjusted estimates for variables ratio > 1.3, Age > 70, CACI; Charlson Age Comorbidity Index, ASA; American Society of Anesthesiologists, active smoking, BMI; Body Mass Index, pT-stage; pathological T-stage post-RC, NAC; neoadjuvant chemotherapy. DFS; Disease-free survival, CSS; Cancer-Specific Survival, OS; Overall survival. * Significant *p*-value in crude estimates suggest association between increased risk of mortality and a ratio > 1.3. ** Significant *p*-value in adjusted estimates age > 70, CACI, ASA active smoking, BMI, pT-stage, or NAC, suggest an association between increased risk of mortality and significant variable. Only variables with values of statistical significance any time in analysis are displayed in table.

## Data Availability

On reasonable request, the corresponding author can make available all codified data from the clinical database used for this study.
